# Models to predict nitrogen excretion from beef cattle fed a wide range of diets compiled from South America

**DOI:** 10.1093/tas/txae072

**Published:** 2024-05-09

**Authors:** Vinícius C Souza, Guilhermo F S Congio, João P P Rodrigues, Sebastião C Valadares Filho, Flávia A S Silva, Luciana N Rennó, Ricardo A Reis, Abmael S Cardoso, Paulo H M Rodrigues, Telma T Berchielli, Juliana D Messana, Cecilia Cajarville, Yury T Granja-Salcedo, Ana L C C Borges, Gilberto V Kozloski, Jaime R Rosero-Noguera, Horacio Gonda, Alexander N Hristov, Ermias Kebreab

**Affiliations:** Department of Animal Science, University of California, Davis, CA 95616, USA; Noble Research Institute LLC, Ardmore, OK 73401, USA; Department of Animal Production, Animal Science Institute, Universidade Federal Rural do Rio de Janeiro, Seropédica, RJ 23897-000, Brazil; Department of Animal Sciences, Universidade Federal de Viçosa, Viçosa, MG 36570-900, Brazil; Department of Animal Sciences, Universidade Federal de Viçosa, Viçosa, MG 36570-900, Brazil; Department of Animal Sciences, Universidade Federal de Viçosa, Viçosa, MG 36570-900, Brazil; Department of Animal Science, Universidade Estadual Paulista, Jaboticabal, SP 14884-900, Brazil; Range Cattle Research and Education Center, University of Florida, Ona, FL 33865, USA; Department of Animal Nutrition and Production, Faculdade de Medicina Veterinária e Zootecnia, Universidade de São Paulo, Pirassununga, SP 13635-900, Brazil; Department of Animal Science, Universidade Estadual Paulista, Jaboticabal, SP 14884-900, Brazil; Department of Animal Science, Universidade Estadual Paulista, Jaboticabal, SP 14884-900, Brazil; Department of Animal Production and Health of Production Systems, Animal Production Institute, Facultad de Veterinaria, Universidad de la República, San José 80100, Uruguay; El Nus Research Center, Corporación Colombiana de Investigación Agropecuaria, San Roque, Antioquia 250047, Colombia; Department of Animal Science, Universidade Federal de Minas Gerais, Belo Horizonte, MG 31270-901, Brazil; Department of Animal Science, Universidade Federal de Santa Maria, Santa Maria, RS 97105-900, Brazil; Faculty of Agricultural Sciences, Universidad de Antioquia, Medellín, Antioquia 050034, Colombia; Department of Animal Nutrition and Management, Faculty of Veterinary Medicine and Animal Science, Swedish University of Agricultural Sciences, Uppsala 75007, Sweden; Department of Animal Science, The Pennsylvania State University, University Park, PA 16802, USA; Department of Animal Science, University of California, Davis, CA 95616, USA

**Keywords:** environmental impacts, grazing cattle, meta-analysis, protein metabolism

## Abstract

The objective of this meta-analysis was to develop and evaluate models for predicting nitrogen (N) excretion in feces, urine, and manure in beef cattle in South America. The study incorporated a total of 1,116 individual observations of N excretion in feces and 939 individual observations of N excretion in feces and in urine (g/d), representing a diverse range of diets, animal genotypes, and management conditions in South America. The dataset also included data on dry matter intake (**DMI**; kg/d) and nitrogen intake (**NI**; g/d), concentrations of dietary components, as well as average daily gain (**ADG**; g/d) and average body weight (**BW**; kg). Models were derived using linear mixed-effects regression with a random intercept for the study. Fecal N excretion was positively associated with DMI, NI, nonfibrous carbohydrates, average BW, and ADG and negatively associated with EE and CP concentration in the diet. The univariate model predicting fecal N excretion based on DMI (model 1) performed slightly better than the univariate model, which used NI as a predictor variable (model 2) with a root mean square error (**RMSE**) of 38.0 vs. 39.2%, the RMSE-observations SD ratio (RSR) of 0.81 vs. 0.84, and concordance correlation coefficient (**CCC**) of 0.53 vs. 0.50, respectively. Models predicting urinary N excretion were less accurate than those derived to predict fecal N excretion, with an average RMSE of 43.7% vs. 37.0%, respectively. Urinary and manure N excretion were positively associated with DMI, NI, CP, average BW, and ADG and negatively associated with neutral detergent fiber concentration in the diet. As opposed to fecal N excretion, the univariate model predicting urinary N excretion using NI (model 10) performed slightly better than the univariate model using DMI (model 9) as predictor variable with an RMSE of 36.0% vs. 39.7%, RSR 0.85 vs. 0.93, and CCC of 0.43 vs. 0.29, respectively. The models developed in this study are applicable for predicting N excretion in beef cattle across a broad spectrum of dietary compositions and animal genotypes in South America. The univariate model using DMI as a predictor is recommended for fecal N prediction, while the univariate model using NI is recommended for predicting urinary and manure N excretion because the use of more complex models resulted in little to no benefits. However, it may be more useful to consider more complex models that incorporate nutrient intakes and diet composition for decision-making when N excretion is a factor to be considered. Three extant equations evaluated in this study have the potential to be used in tropical conditions typical of South America to predict fecal N excretion with good precision and accuracy. However, none of the extant equations are recommended for predicting urine or manure N excretion because of their high RMSE, and low precision and accuracy.

## Teaser

The models developed in this study to predict N excretion in beef cattle can help to improve the environmental and economic sustainability of beef production systems in South America.

## INTRODUCTION

The ability of ruminants to convert human inedible fibrous compounds into highly valuable animal proteins is of great importance in terms of global human food production. This is especially crucial given the rapidly increasing global population and the growing demand for plant resources suitable for human consumption (Salter, 2017). However, increased demand for animal-based products such as meat and milk is expected to have a negative environmental impact because of greenhouse gas (**GHG**) emissions associated with animal production, notably enteric methane and nitrous oxide (FAO, 2013). Nitrogen (**N**) excreted by ruminants can be converted to nitrous oxide, a GHG with a global warming potential approximately 273 times greater than CO_2_ (IPCC, 2021). In addition, ammonia volatilized from ruminant excreta has negative consequences for human health, including respiratory diseases and premature mortality (McCubbin et al., 2002). Nitrate leaching from cattle excreta can also contribute to N_2_O emissions if nitrate is denitrified within surface waters or humid soils (Cai and Akiyama, 2016).

In general, N use efficiency in ruminants is low, ranging from 11% to 40% (Dijkstra et al., 2011). In growing beef cattle, urine is the route of approximately 60% to 80% of total N excreted, while 20% to 40% is excreted in feces (Dong et al., 2014). The proportion of N excreted in urine or feces is influenced by N intake (**NI**), apparent N digestibility, and dietary crude protein (**CP**) content (Souza et al., 2021). The large variation observed in N use efficiency in beef cattle represents an opportunity to decrease N excretion through diet manipulation. Such reductions not only yield environmental advantages but also confer economic benefits associated with a reduction in the purchase of protein feed ingredients. Because measuring N excretion under farm conditions is not feasible from a logistic and economic standpoint, models that can accurately predict N excretion using variables that are more easily obtained in beef production systems are needed. With these models, beef producers can predict N excretion in beef cattle and evaluate the N use efficiency in their operations. A variety of models exist in the literature to predict nitrogen excretion in beef cattle (Waldrip et al., 2013; Dong et al., 2014; Reed et al., 2015; Bougouin et al., 2022). These models were developed using animals and diets in temperate conditions typical of Europe and North America. However, they might not fully represent dietary conditions, animal genotypes, and management approaches adopted by beef producers in tropical conditions encountered in South America (Congio et al., 2023), all of which are expected to influence N excretion. Bougouin et al. (2022) recently conducted a meta-analysis to develop models to predict N excretion in beef cattle using an intercontinental database. Although the authors used data collected from Brazil, their database included mainly data from North America and Europe obtained in conditions that may not represent beef production systems in South America.

The objective of this meta-analysis was to develop and evaluate models for predicting N excretion in feces, urine, and total manure (urine + feces, hereafter mentioned only as manure) in beef cattle using an extensive database composed of individual N excretion records representing a diverse range of diets, animal genotypes, and management conditions in South America.

## MATERIALS AND METHODS

Animal Care and Use Committee approval was not specifically required for this analysis because approvals were obtained by coauthors during the original studies.

### Database Description

This study is part of the Global Network project and the Feed and Nutrition Network, which is an activity of the Livestock Research Group of the Global Research Alliance for Agricultural Greenhouse Gases (https://globalresearchalliance.org). The database used in this study was collated during the Latin America Methane Project (Congio et al., 2022), using individual observations (raw data) provided by collaborators from South America (Brazil, Colombia, Argentina, and Uruguay). The following information was included in the database: 1) fecal and urinary N excretions (g/d) measured by either total feces and urine collection or spot sampling associated with marker methods, 2) dry matter intake (**DMI**; kg/d) and NI (g/d), 3) dietary nutrient concentrations (g/kg DM) such as CP, neutral detergent fiber (**NDF**), ether extract (**EE**), nonfibrous carbohydrates (**NFC**), and forage proportion (**FP**, % DM), and 4) performance (**PF**) such as average daily gain (**ADG**, g/d) and average body weight (**BW**, kg). Because we only had dietary starch concentration for 220 observations, we estimated the dietary NFC concentration using the following equation: NFC=100−(CP+NDF+EE+Ash). The initial database was composed of 1,116 and 939 observations of N excretion by feces and urine, respectively. A list of the studies included in the database is provided in Supplementary Table S1.

### Data Preselection for Model Development

Low DMI values (<1% of BW) were removed from the analysis because they would be unrealistic within a practical farming context (*n* = 30). For models predicting urinary and manure N excretion, we removed 117 observations from 5 studies that presented inconsistencies in N balance. Detailed information on the reason for the exclusion of each study is presented in Supplementary Table S2. An additional screening was carried out to identify improbable observations. Observations where N excretion in urine, expressed in percentage of N intake, where below 10% or above 60% were also excluded using the range of values found in several reports in the literature as criteria (Waldrip et al., 2013; Dong et al., 2014; Angelidis et al., 2019; Bougouin et al., 2022). This procedure resulted in the exclusion of 75 observations. In addition, we have excluded 84 observations where N retention was negative, which suggested problems in urinary N excretion determination since most of these observations were from animals that were gaining weight. Finally, we removed one observation of ADG (2.86 kg/d) and one observation of DMI (20.9 kg/d) as these values are unrealistic for beef cattle averaging 353 kg of BW (BR Corte, 2023).

Fecal, urinary, and manure N data were analyzed for outliers by using the boxplot function as well as the interquartile range (**IQR**) method (Zwillinger and Kokoska, 2000). The IQR method aims identify outliers by setting up a limit outside of Q1 and Q3. Any values that fall outside of this limit are considered outliers (Supplementary Fig. S1). The factor of 1.5 was used in constructing markers to identify outliers (Bougouin et al., 2022). As a result, we removed 14, 7, and 14 outliers for fecal, urinary, and manure N excretions, respectively.

After data cleaning (described below), the resultant database was composed of 1,070 and 628 observations of N excretion in feces and in urine, respectively from beef cattle (Nellore [61.1%], nonspecified crossbreeds [7.00%], Holstein [5.42%], Hereford [4.39%], Holstein × Zebu [2.24%], Nellore × Angus [2.99%], Brangus [1.31%], Aberdeen Angus × Hereford [2.43%], Guzera [1.12%], and Gyr [0.56%]), were obtained from 39 in vivo experiments. The data were provided by collaborators from Universidade Estadual Paulista, Brazil (10 experiments and 365 individual observations), Universidade Federal de Viçosa, Brazil (11 experiments and 333 individual observations), Universidade de São Paulo, Brazil (7 experiments and 177 individual observations), Universidad de la República, Uruguay (3 experiments and 73 individual observations), Universidade Federal de Minas Gerais, Brazil (3 experiments and 56 individual observations), Universidade Federal de Santa Maria, Brazil (2 experiments and 32 individual observations), Universidad de Antioquia, Colombia (2 experiments and 24 individual observations), Universidad Nacional del Centro de la Provincia de Buenos Aires, and Argentina (1 experiment and 20 individual observations). In this database, 72.8% of the data were from confined animals, while grazing animals represented 27.2% of the data. The main forage sources used for grazing animals were *Urochloa brizantha* (75.9% of the data), *Urochloa decumbens* (17.2%), and *Avena sativa* (6.87%). For confined animals, the main forage sources were corn silage (66.0%), tifton 85 hay (12.5%), sorghum silage (5.42%), fresh chopped sugar cane (4.24%), grass silage (3.08%), *Trifolium repens* and *Lolium multiflorum* fresh forages (2.95%), fresh *Avena strigosa* (2.05%), *Digitaria decumbens* hay (1.80%), fresh legumes (1.16%), and Snaplage (0.90%). Measurements of N excretion in feces and urine were conducted using total fecal and urine collection (46.6% of the data) or marker and spot sampling approaches (53.4% of the data). Males (intact and castrated) represented 56.7% of the data and females 43.3%.

### Model Derivation

All data were analyzed using R version 4.1.1 (R Core Team, 2021). Models were derived using linear mixed-effects regression with a random intercept for study (St-Pierre, 2001) according to the following model structure:


$$\begin{array}{l} Y_{ij}=\beta_{0}+\beta_{1}X_{ij1}+\beta_{2}X_{ij2}+\cdots +\beta_{k}X_{ijk}+S_{i}+\varepsilon_{ij}, \end{array}$$

where *Y*_*ij*_ denotes the *j*-th response variable of fecal, urinary, or manure N excretions (g/d) from the *i*-th study; *β*_*0*_ denotes the fixed effect of intercept; *X*_*ij1*_ to *X*_*ijk*_ denote the fixed effects of predictor variables and *β*_*1*_ to *β*_*k*_ are the corresponding slopes; *S*_*i*_ and *ɛ*_*ij*_ are the random effect of study and residual error, respectively.

Error normality was checked at each step of the model derivation process through the evaluation of residual plots. Models were derived using the *lme* function from the *nlme* R package to solve parameter estimates (Pinheiro et al., 2021).

For model derivation purposes, several approaches were explored to split the data in order to improve model performance, including models specific to the dietary CP (g/kg DM; CP < 90, 90 < CP ≤ 150, and 150 < CP) or forage levels (% DM; FP < 30, 30 < FP < 70, 70 ≤ FP < 100, and FP = 100, which refers to forage diets + mineral supplements), and the housing system (feedlot or grazing). We also split the data based on the method used to determine urine and fecal production to explore the effects of spot sampling methods on the ability of the models to predict these response variables. Eight models were derived using sets of data that included univariate models such as DMI and NI, dietary composition (**DC**) variables, and PF variables (ADG and average BW) individually and in combinations to understand factors that drive variability in the three response variables: fecal N excretion, urinary N excretion, and manure N excretion. Dietary composition included CP, NDF, EE, NFC, and FP. We have not included ADG as an explanatory variable for the grazing dataset because there were only 20 observations from 2 studies. Variables used for model development and their summary statistics are given in Table 1.

**Table 1.** Summary statistics of variables used for model development for each response variable

Variables^1^	Fecal N					Urine N					Manure N				
	*n* ^2^	Mean	SD	Min^3^	Max^3^	*n*	Mean	SD	Min	Max	*n*	Mean	SD	Min	Max
*Intake*															
DMI, kg/d	1,070	6.98	2.41	1.69	15.2	614	6.32	2.04	1.95	13.4	607	6.28	2.00	1.95	13.4
NI, g/d	1,067	150	56.6	26.5	370	614	136	47.6	26.5	370	607	135	44.6	26.5	340
*Diet composition*															
CP, g/kg DM	1,067	135	28.4	22.8	287	614	137	29.5	22.8	287	607	136	29.1	22.8	287
NDF, g/kg DM	1,047	415	141.5	181	753	594	411	143	181	736	587	410	144	181	736
EE, g/kg DM	860	31.8	12.1	10.3	89.2	451	31.7	9.36	11.2	62.0	446	31.8	9.38	11.2	62.0
NFC, g/kg DM	775	374	163.3	6.60	605	380	395	174	6.60	605	378	397	174	6.60	605
FP, %	1,020	62.0	25.8	17.0	100	583	60.8	27.2	17.0	100	578	60.6	27.2	17.0	100
*Performance*															
ADG, kg/d	303	0.854	0.428	0.00	2.15	195	0.822	0.377	0.00	1.64	190	0.816	0.373	0.00	1.64
Average BW, kg	991	355	129.1	110	784	557	302	80.6	110	572	552	302	80.5	110	572
*N excretion*															
Fecal N, g/d	1,070	45.1	21.0	6.32	105						607	38.8	17.5	7.14	97.5
Urine N, g/d						614	46.2	18.6	4.20	95.2	607	45.9	18.5	4.20	102
Manure N, g/d											607	84.7	26.6	19.4	157
N retention, g/d						614	50.5	31.5	0.428	209					
NUE, %						614	35.2	13.5	0.45	72.4					

^1^DMI, dry matter intake; NI, nitrogen intake; CP, crude protein, NDF, neutral detergent fiber; EE, ether extract; NFC, nonfibrous carbohydrates; FP, forage proportion; ADG, average daily gain; BW, body weight.

^2^Number of observations.

^3^Min, minimum; Max, maximum.

Models were generated using a multistep selection approach as described by van Lingen et al. (2019). The initial selection of variables was based on their biological relevance concerning their correlation with N excretion in feces or urine. Briefly, a backward selection approach was applied whereby only covariates selected in a prior step could be selected for the subsequent step. The model selection procedure stopped when the selected covariates were the same as the ones selected in the previous step. Bayesian information criterion values were calculated for all fitted models, and those with the smallest values were chosen (James et al., 2014). The multicollinearity among covariates in multiple regression models was verified using the variance inflation factor (Zuur et al., 2010) and models were chosen when all covariates had values lower than three.

### Evaluation of the Developed Models and Extant Equations

A k-fold cross-evaluation was performed to evaluate the predictive ability of fitted prediction models at different levels, where K was equal to the number of studies included in the database (James et al., 2014). Briefly, studies (folds) were sequentially taken as the testing set for model evaluation, whereas those that remained were considered as the training set for model fitting. Thus, each individual fold was treated as an evaluation set, where the prediction of N excretion of each fold was calculated using the model that was fitted from the remaining folds (Bougouin et al., 2022). We also evaluated 25 extant equations predicting N excretion in feces, urine, and manure in beef cattle (Waldrip et al., 2013; Dong et al., 2014; Reed et al., 2015; Angelidis et al., 2019; Bougouin et al., 2022). The choice of the extant models was based on predictive performance in the original publications and the availability of predictor variables in our database. Model quality was assessed based on the root mean square error (**RMSE**), expressed a percentage of the observed daily N excretion, RMSE-observations standard deviation ratio (**RSR**) of observed values, and concordance correlation coefficient (**CCC**; Lin, 1989). The mean squared prediction error was decomposed into mean (**MB**) and slope bias (**SB**) deviations to identify systematic biases, as performed by Bougouin et al. (2022). We also included the determination coefficient (*R*^2^) in the cross-validation to determine how much of the variation in the response variables was explained by the developed models.

The RSR was used to compare the performance of models developed from different datasets. This approach considers standardized model performance relative to the variability in observations from different datasets (Moriasi et al., 2007). Smaller RSR (<1) indicates superior performance, given the variability of observations. Lin’s CCC was used to evaluate the agreement between the best-fit line and the identity line (*y* = *x*). A CCC closer to 1, indicates better model performance. Coefficients were obtained using predicted values that were calibrated for study effects. For final comparisons of models using different derivation data sets, CCC was the main tool used for model comparison. Plots of the observed versus predicted values for each model were developed using the *ggplot* function from the *ggplot2* R package for a visual evaluation of model quality.

## RESULTS

Descriptive statistics of the explanatory and response variables are given in Table 1. None of the approaches tested to subset our database (CP or forage levels, housing system, and method used to determine fecal and urine output) resulted in significant improvements in model performance (results not presented). Thus, only one set of models for each response variable is presented.

### Evaluation of Extant Equations

Results of the performance evaluation of extant models predicting N excretion in feces, urine, and manure are included in Table 2. For the extant equations predicting fecal N excretion, the equations from Dong et al. (2014) and Angelidis et al. (2019) stood out, presenting a similar and good predictive performance. Specifically, the equation from Dong et al. (2014) was ranked first with a RMSE of 38.0%, good precision (MB = 0.25%), and accuracy (SB = 0.49%) when NI was used as a predictor. However, when DMI was used as a predictor, the best equation was from Angelidis et al. (2019), which has a RMSE of 37.1, MB of 0.67%, and SB of 1.59%. The remaining extant equations evaluated to predict fecal N excretion all presented high MB and/or SB, which was considered unacceptable. In contrast, any of the equations evaluated to predict urine and manure N excretion were considered acceptable because of their high RMSE, low precision (high MB), and/or accuracy (high SB).

**Table 2.** Performance evaluation of extant equations to predict N excretion in feces and urine in beef cattle using the South America database

Reference	Prediction equation^1^	Model performance^2^						*R* ^2^
		*n*	RMSE, %	RSR	Mean bias, %	Slope bias, %	CCC	
*Fecal excretion, g/d*								
Bougouin et al. (2022)	5.03 + 6.49 × DMI	1,047	39.1	0.83	9.98	1.83	0.57	0.39
Bougouin et al. (2022)	13.5 + 0.24 × NI	1,047	39.2	0.83	6.61	0.37	0.52	0.35
Bougouin et al. (2022)	−37.7 + 6.27 × DMI + 0.17 × CP + 0.06 × NDF	1,047	41.2	0.88	24.1	2.38	0.58	0.44
Waldrip et al. (2013)	24.28 + 0.154 × NI	1,047	39.2	0.83	1.85	4.70	0.41	0.35
Dong et al. (2014)	15.82 + 0.20 × NI	1,047	38.0	0.81	0.25	0.49	0.49	0.35
Reed et al. (2015)	0.506 + 0.352 × NI	1,047	45.3	0.96	16.9	13.2	0.55	0.35
Angelidis et al. (2019)	21.94 + 0.158 × NI	1,047	38.7	0.82	0.14	4.24	0.43	0.35
Angelidis et al. (2019)	1.63 + 6.378 × DMI	1,047	37.1	0.79	0.67	1.59	0.59	0.39
*Urinary N excretion, g/d*								
Bougouin et al. (2022)	22.4 + 7.56 × DMI	557	65.8	1.65	62.0	7.25	0.20	0.17
Bougouin et al. (2022)	12 + 0.38 × NI	557	57.0	1.42	48.1	13.5	0.31	0.22
Bougouin et al. (2022)	−63 + 0.67 × CP + 0.10 × BW	557	59.0	1.47	28.0	28.7	0.18	0.06
Bougouin et al. (2022)	−96.8 + 6.81 × DMI + 0.69 × CP + 0.09 × NDF	537	95.4	2.34	55.0	28.0	0.12	0.06
Waldrip et al. (2013)	−21.18 + 0.56 × NI	557	57.1	1.43	13.5	48.3	0.40	0.22
Dong et al. (2014)	−14.12 + 0.51 × NI	557	53.8	1.34	15.9	41.1	0.41	0.22
Reed et al. (2015)	6.80 + 0.405 × NI	557	55.4	1.39	41.4	18.0	0.34	0.22
Angelidis et al. (2019)	−26.49 + 0.597 × NI	557	59.6	1.49	11.8	53.3	0.40	0.22
Angelidis et al. (2019)	−10.47 + 11.3 × DMI	557	57.5	1.44	25.3	34.5	0.33	0.17
*Manure N excretion, g/d*								
Bougouin et al. (2022)	33.3 + 13.60 × DMI	532	47.3	1.50	75.6	4.26	0.41	0.55
Bougouin et al. (2022)	23.1 + 0.63 × NI	532	37.0	1.18	62.0	7.42	0.54	0.58
Bougouin et al. (2022)	−18.8 + 0.61 × CP + 0.07 × NDF + 0.06 × BW	532	48.4	1.54	46.2	13.7	0.12	0.04
Bougouin et al. (2022)	−139.4 + 14.0 × DMI + 0.87 × CP + 0.14 × NDF	532	63.3	2.01	58.9	24.9	0.31	0.34
Reed et al. (2015)	6.916 + 0.759 × NI	532	40.6	1.29	57.1	17.6	0.54	0.58
Angelidis et al. (2019)	−5.681 + 0.761 × NI	532	31.2	0.99	26.6	30.3	0.67	0.58
Angelidis et al. (2019)	−14.42 + 18.27 × DMI	532	35.5	1.13	29.3	35.2	0.62	0.55
Angelidis et al. (2019)	−15.77 + 0.757 × NI + 0.020 × BW + 0.105 × FP	532	32.9	1.05	30.6	31.3	0.65	0.58

^1^DMI, dry matter intake (kg/d); NI, nitrogen intake (g/d); CP, crude protein (g/kg DM); BW, body weight (kg); FP, forage proportion (% DM).

^2^Model evaluation metrics. RMSE, root mean square error, expressed as a percentage of observed response variable; RSR, RMSE-observations standard deviation ratio; CCC, concordance correlation coefficient; *R*^2^: determination coefficient.

### Fecal N Excretion

**Figure 1.**
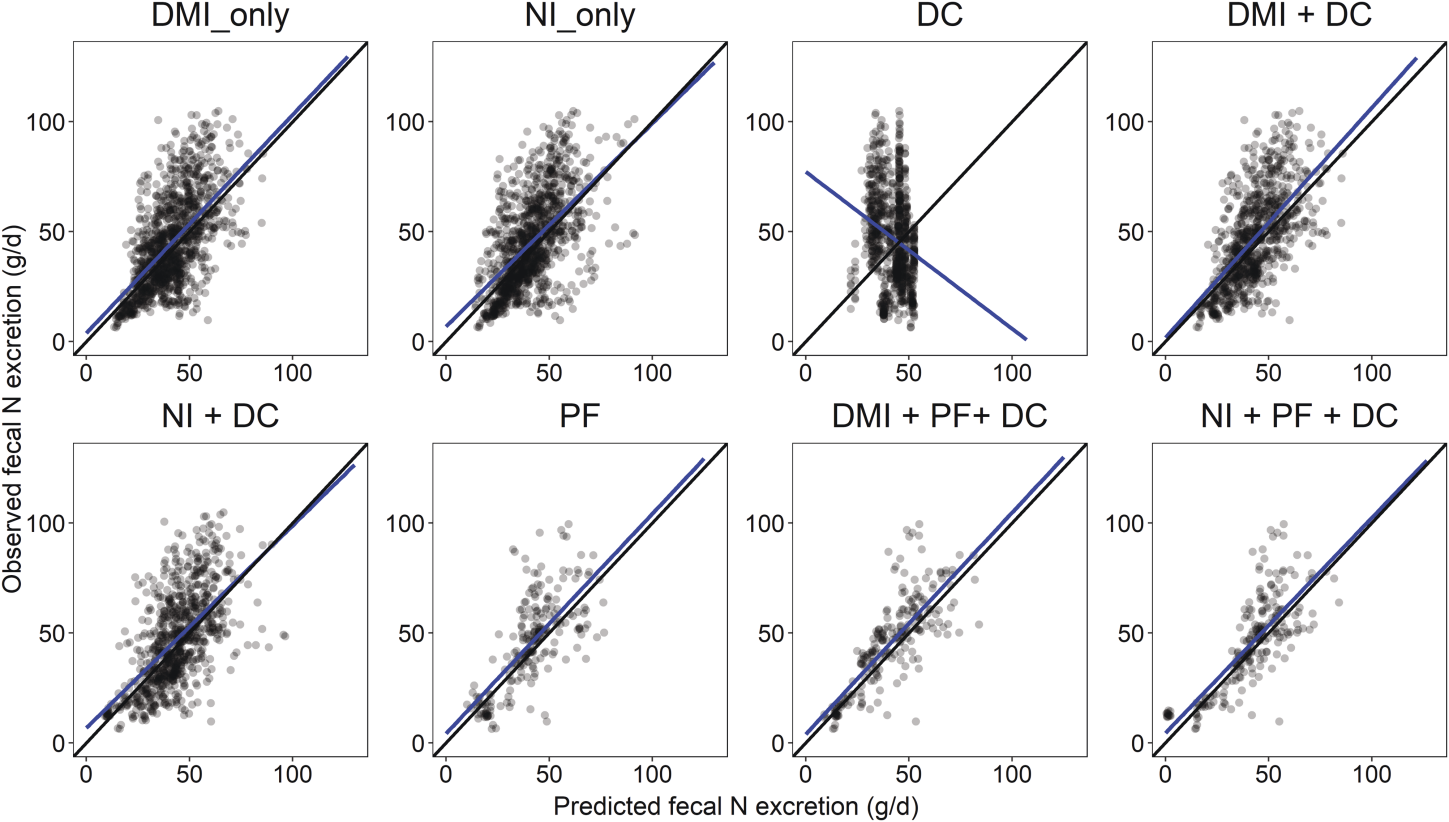
Relationship between observed and predicted fecal N excretion (g/d). The black solid line is the identity line (*y* = *x*), and the blue solid line is the fitted regression line for the relationship between observed and predicted values. Abbreviations: DMI, dry matter intake; NI, nitrogen intake; DC, dietary composition variables; PF, performance variables.

Models to predict fecal N excretion are given in Table 3. Plots of the relationship between observed and predicted values from each model developed are available in Fig. 1. Fecal N excretion was positively associated with DMI, NI, NFC, average BW, and ADG and negatively associated with dietary concentrations of EE and CP. The univariate model predicting fecal N excretion based on DMI (model 1) performed slightly better than the univariate model which used NI as a predictor variable (model 2) with an RMSE of 38.0% vs. 39.2% and RSR of 0.81 vs. 0.84, CCC of 0.53 vs. 0.50, and *R*^2^ of 0.36 vs. 0.33, respectively. The model derived using only DC variables (model 3), which included EE and NFC, performed poorly with increased RMSE, RSR, MB, SB, and reduced CCC and *R*^2^ when compared to univariate models (models 1 and 2), as evidenced in the DC plot in Fig. 1. Combining nutrient intakes (DMI or NI) with DC variables resulted in minor or no improvements in the models predicting fecal N excretion (models 4 and 5) compared to simpler univariate models (models 1 and 2). The inclusion of PF variables (ADG and/or average BW) in models 6, 7, and 8 resulted in an improvement in RMSE, CCC, and *R*^2^ relative to univariate models or DMI + DC and NI + DC models; however, MB also increased significantly. In addition, models derived using nutrient intakes and DC variables with performance variables (models 7 and 8) performed better than model 6, which included only PF variables.

**Table 3.** Models predicting fecal N excretion (g/d per animal) according to different categories and performance evaluation

Model	Category^1^	Prediction equation^2^	Model performance^3^						
			*n*	RMSE, %	RSR	Mean bias, %	Slope bias, %	CCC	*R* ^2^
1	DMI_only	4.64* (±2.42) + 5.29*** (±0.20) × DMI	1,067	38.0	0.81	4.15	0.00	0.53	0.36
2	NI_only	7.29*** (±2.39) + 0.23*** (±0.01) × NI	1,067	39.2	0.84	4.17	0.31	0.50	0.33
3	DC	33.4*** (±4.96) − 0.21*** (±0.06) × EE + 0.04*** (±0.01) × NFC	775	51.9	1.15	4.54	24.0	−0.13	0.05
4	DMI + DC	13.6*** (±3.16) + 5.14*** (±0.24) × DMI—0.22*** (±0.05) × EE	775	35.9	0.80	4.79	0.12	0.55	0.40
5	NI + DC	39.9*** (±4.19) + 0.25*** (±0.01) × NI—0.21*** (±0.02) × CP—0.21*** (±0.04) × EE	775	37.0	0.82	3.59	0.39	0.53	0.35
6	PF	−6.72* (±3.60) + 0.09*** (±0.01) × average BW + 14.9*** (±2.27) × ADG	218	34.1	0.74	7.35	3.54	0.64	0.49
7	DMI + DC + PF	−3.09 (±2.63) + 4.94*** (±0.37) × DMI + 6.04*** (±2.17) × ADG	218	29.5	0.64	10.1	0.01	0.75	0.63
8	NI + DC + PF	19.4** (±9.36) + 0.24*** (±0.02) × NI + 5.57** (±2.28) × ADG—0.16*** (±0.06) × CP	218	30.0	0.65	6.80	0.07	0.74	0.60

^1^DMI: Dry matter intake (kg/d), NI: Nitrogen intake (g/d), DC: Dietary composition variables, and PF: Performance variables.

^2^EE: ether extract (g/kg DM), NFC: nonfibrous carbohydrates (g/kg DM), CP: crude protein (g/kg DM), FP: forage proportion (% DM), BW: body weight (kg), and ADG: average daily gain (kg/d).

^3^Model evaluation metrics obtained from the k-fold cross-evaluation. RMSE: Root mean square error, expressed as a percentage of observed daily N excretion in feces, RSR: RMSE-observations standard deviation ratio, CCC: Concordance correlation coefficient, and *R*^2^: determination coefficient.

***P-value ≤ 0.01, **P-value ≤ 0.05, *P-value ≤ 0.10. Parameters without asterisk indicate a *P*-value > 0.10.

### Urinary N Excretion

**Figure 2.**
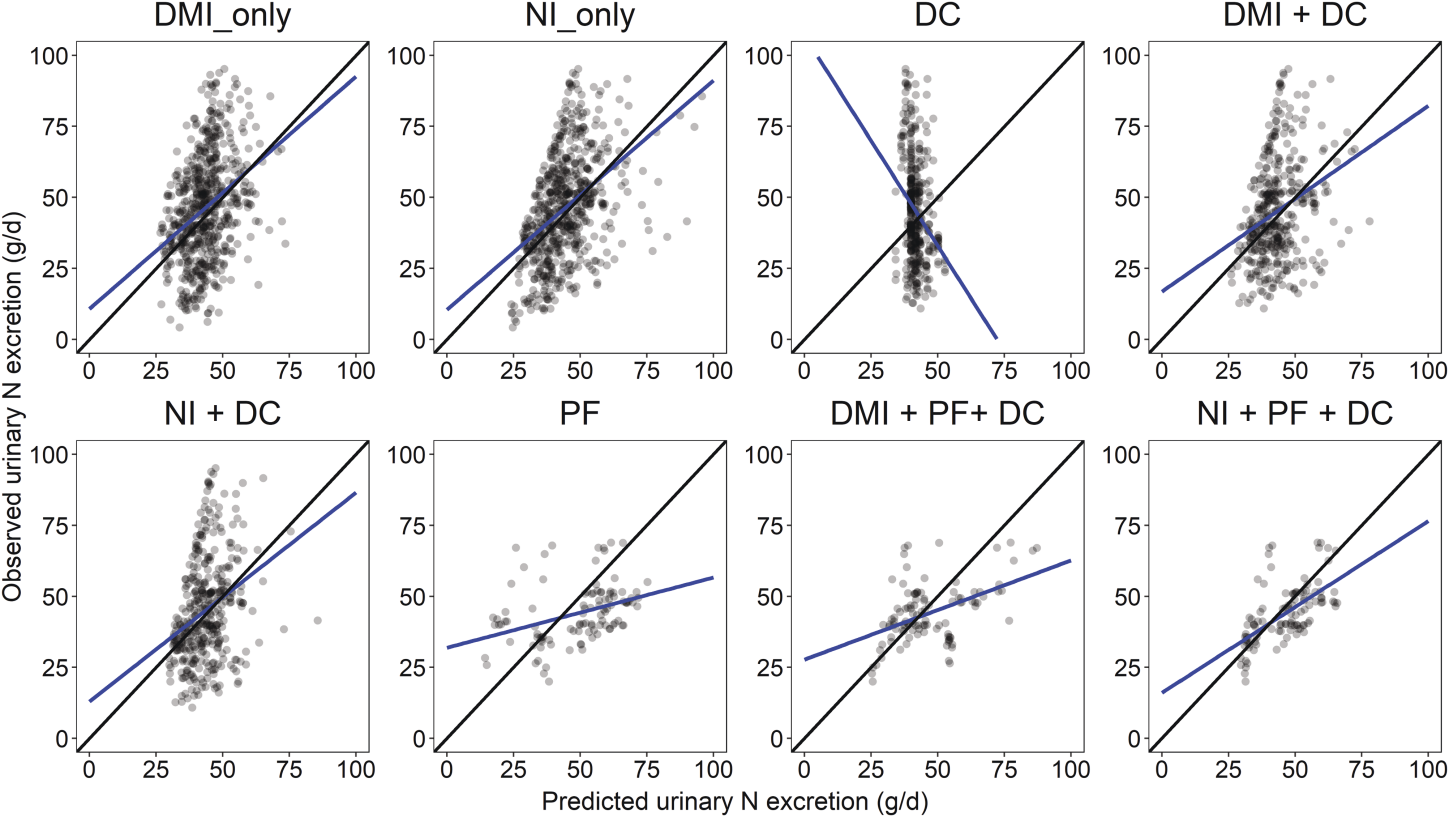
Relationship between observed and predicted urinary N excretion (g/d). The black solid line is the identity line (*y* = *x*), and the blue solid line is the fitted regression line for the relationship between observed and predicted values. Abbreviations: DMI, dry matter intake; NI, nitrogen intake; DC, dietary composition variables; PF: performance variables.

Models to predict urinary N excretion are given in Table 4. Plots of the relationship between observed and predicted urinary N excretion are available in Fig. 2. Models predicting urinary N excretion were slightly more accurate than those derived to predict fecal N excretion with an average RMSE of 35.3% vs. 37.0%, respectively. Urinary N excretion was positively associated with DMI, NI, CP, and average BW. In contrast to fecal N excretion, the univariate model predicting urinary N excretion using NI (model 10) performed slightly better than the one using DMI (model 9) as predictor variable with an RMSE of 36.5% vs. 38.2%, RSR 0.91 vs. 0.95, CCC of 0.37 vs. 0.25, *R*^2^ of 0.19 vs. 0.12, respectively. The DC model predicting urinary N excretion, which only included CP, performed poorly in cross evaluation with increased RMSE, RSR, MB, SB, and reduced CCC and *R*^2^ when compared to univariate models (models 9 and 10), as evidenced in the DC plot in Fig. 2, similar to the DC model predicting fecal N excretion. Combining nutrient intakes (DMI or NI) with DC variables resulted in no improvements in predictive performance in the models predicting urinary N excretion (models 12 and 13) compared to simpler univariate models (models 9 and 10). As expected, models derived only using DC (model 11), PF variables (model 14), or the combination of nutrient intakes with DC and PF variables (models 15 and 16) resulted in considerable MB and/or SB (Table 4; Fig. 2).

**Table 4.** Models predicting urinary N excretion (g/d per animal) according to different categories and performance evaluation

Model	Category^1^	Prediction equation^2^	Model performance^3^						
			*n*	RMSE, %	RSR	Mean bias, %	Slope bias, %	CCC	*R* ^2^
9	DMI_only	19.2*** (±3.21) + 3.83*** (±0.32) × DMI	614	38.2	0.95	2.55	0.67	0.25	0.12
10	NI_only	15.7*** (±2.83) + 0.21*** (±0.01) × NI	614	36.5	0.91	1.24	1.35	0.37	0.19
11	DC	29.7*** (±5.36) + 0.09*** (±0.03) × CP	380	43.1	1.08	2.87	17.0	−0.09	0.07
12	DMI + DC	−6.35 (±5.83) + 3.90*** (±0.35) × DMI + 0.19*** (±0.03) × CP	380	39.0	0.97	1.10	2.57	0.22	0.09
13	NI + DC	20.1*** (±3.79) + 0.18*** (±0.02) × NI	380	38.5	0.96	0.68	1.32	0.22	0.09
14	PF	0.77 (±7.31) + 0.15*** (±0.01) × average BW	106	36.1	1.53	10.3	53.4	0.32	0.13
15	DMI + DC + PF	−18.5 (±12.6) + 4.73*** (±0.42) × DMI + 0.26*** (±0.07) × CP	106	29.7	1.26	4.41	46.4	0.43	0.21
16	NI + DC + PF	18.3*** (±3.85) + 0.21*** (±0.01) × NI	106	21.6	0.91	5.97	18.9	0.59	0.37

^1^DMI, dry matter intake (kg/d); NI, nitrogen intake (g/d); DC: dietary composition variables; PF, performance variables.

^2^NDF, neutral detergent fiber (g/kg DM); CP, crude protein (g/kg DM); BW, body weight (kg); ADG: average daily gain (kg/d).

^3^Model evaluation metrics obtained from the k-fold cross-evaluation. RMSE, root mean square error, expressed as a percentage of observed daily N excretion in urine; RSR, RMSE-observations standard deviation ratio; CCC, concordance correlation coefficient; *R*^2^: determination coefficient. ***P-value ≤ 0.01, **P-value ≤ 0.05, *P-value ≤ 0.10. Parameters without asterisk indicate a *P*-value > 0.10.

### Manure N Excretion

**Figure 3.**
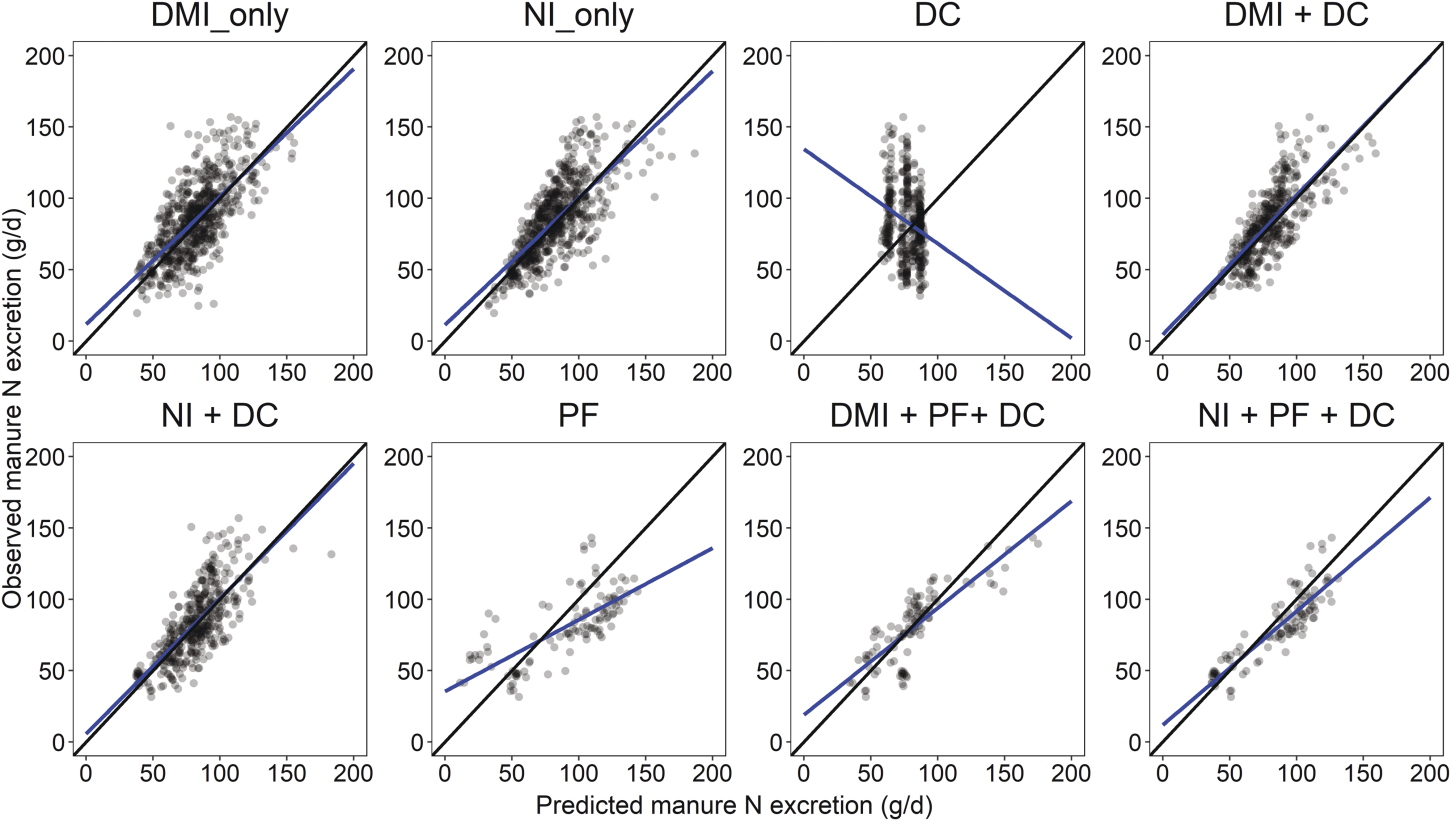
Relationship between observed and predicted manure N excretion (g/d). The black solid line is the identity line (*y* = *x*), and the blue solid line is the fitted regression line for the relationship between observed and predicted values. Abbreviations: DMI, dry matter intake; NI, nitrogen intake; DC, dietary composition variables; PF: performance variables.

Models to predict manure N excretion are given in Table 5. Plots of the relationship between observed and predicted manure N excretion for each model developed are available in Fig. 3. Overall, models predicting manure N excretion performed better than models predicting either fecal or urinary N excretion by showing a lower RMSE and greater CCC and *R*^2^. Manure N excretion was positively associated with DMI, NI, CP, NFC, average BW, and ADG and negatively correlated with NDF content. The univariate model predicting manure N excretion based on NI (model 18) performed slightly better than the univariate model, which used DMI as a predictor variable (model 17) with an RMSE of 21.7% vs. 23.2%, RSR 0.69 vs. 0.74, and CCC of 0.72 vs. 0.66, and *R*^2^ of 0.54 vs. 0.47, respectively. Similar to models predicting fecal N and urinary N excretions, the DC model (model 19), which only included NFC as a predictor, performed poorly with the highest RMSE (35.6%) and the lowest CCC (−0.15). Similar to the fecal and urinary N excretion models, models predicting manure N excretion based on PF or the combination of nutrient intakes with DC and PF resulted in models with greater MB and/or SB relative to univariate models.

**Table 5.** Models predicting manure N excretion (g/d per animal) according to different categories and performance evaluation for the complete dataset.

Model	Category^1^	Prediction equation^2^	Model performance^3^						
			N	RMSE, % mean	RSR	Mean bias, % MSE	Slope bias, % MSE	CCC	*R* ^2^
17	DMI_only	17.9*** (±3.60) + 10.2*** (±0.41) × DMI	607	23.2	0.74	2.56	1.21	0.66	0.47
18	NI_only	17.1*** (±2.97) + 0.49*** (±0.02) × NI	607	21.7	0.69	1.35	1.74	0.72	0.54
19	DC	58.4 (±8.94)*** + 0.05*** (±0.02) × NFC	378	35.6	1.17	3.83	27.1	−0.15	0.06
20	DMI + DC	−14.1** (±6.64) + 10.4*** (±0.42) × DMI + 0.23*** (±0.04) × CP	378	20.5	0.67	2.05	0.08	0.72	0.56
21	NI + DC	45.6*** (±5.50) + 0.49*** (±0.02) × NI—0.21*** (±0.03) × CP	378	20.8	0.68	0.64	0.36	0.71	0.54
22	PF	−30.2*** (±11.7) + 0.34*** (±0.03) × average BW + 11.9*** (±4.22) × ADG	106	32.7	1.06	12.4	44.9	0.64	0.51
23	DMI + DC + PF	−18.0 (±15.7) + 11.8*** (±0.47) × DMI + 0.37*** (±0.08) × CP—0.07*** (±0.03) × NDF	106	19.8	0.64	1.09	18.0	0.81	0.66
24	NI + DC + PF	−10.7 (±7.63) + 0.58*** (±0.02) × NI + 0.05** (±0.02) × NFC	106	17.4	0.56	15.7	16.0	0.86	0.78

^1^DMI, dry matter intake (kg/d); NI, nitrogen intake (g/d); DC: dietary composition variables; PF: performance variables.

^2^NFC, nonfibrous carbohydrates (g/kg DM); CP, crude protein (g/kg DM); NDF, neutral detergent fiber content (g/kg DM); ADG, average daily gain (kg/d); BW, body weight (kg).

^3^Model evaluation metrics obtained from the k-fold cross-evaluation. RMSE, root mean square error, expressed as a percentage of observed daily N excretion in manure; RSR, RMSE-observations standard deviation ratio; CCC, concordance correlation coefficient; *R*^2^: determination coefficient.

***P-value ≤ 0.01, **P-value ≤ 0.05, *P-value ≤ 0.10. Parameters without asterisk indicate a *P*-value > 0.10.

## DISCUSSION

### Overview and Limitations

The equations developed in this study were designed for application in beef cattle that are fed forage-based diets, particularly focusing on the context of South America. These equations are well-suited for Nellore cattle, which comprised 61.1% of the database. High-concentrate diets (≥70% of concentrate feeds, DM basis) represented only 19.3 (*n* = 207), 30.6 (*n* = 188), and 30.6% (*n* = 186) of the data used to develop models to predict fecal, urinary, and manure N excretions, respectively. Although forage proportion was not significant (*P* > 0.10) in any of the models that included DC variables, caution should be taken when applying these equations to feedlot systems where high-concentrate diets are used (Pinto and Millen, 2019). Because of the low proportion of high-grain diets in this dataset, validation of the equations in beef cattle fed such diets, commonly used in feedlots, should be done prior to the application of these equations.

The models developed in this study using NI had lower performance compared with previous studies predicting N excretion in beef cattle (Waldrip et al., 2013; Reed et al., 2015; Bougouin et al., 2022). This may be attributed to the broader range of diets and animal genotypes included in the database used in this study (Bougouin et al., 2022) or perhaps due to challenges related to measuring N excretion, which can increase the prediction error. Although N balance studies in ruminants have been performed for more than 150 yr, it is still extremely challenging to obtain accurate measures of fecal and urinary N excretions (Hristov et al., 2019). Our models indicate problems with the determination of the variables used to predict N excretion in urine, such as nutrient intakes, diet composition, or with the application of the methods available for measuring urinary N excretion, or both. It is also important to note that the problems associated with the application of spot sampling methods used for measuring N excretion in urine might be only part of the problem with urine N excretion measurements in our dataset because excluding studies that performed urine spot sampling did not result in considerable improvements in our models.

### Model Performance and Key Predictors

Only three equations predicting fecal nitrogen excretion were recommended from the extant equations evaluated in this study. However, no existing equation was found to be adequate for predicting urine nitrogen or manure nitrogen in beef cattle raised in tropical conditions typical of South America. This highlights the need for the development of new equations specifically targeted to South America. In this study, the univariate models based on DMI or NI outperformed or performed similarly to more complex models including DC and PF variables. This finding contrasts with previous meta-analysis aimed at predicting N excretion in beef cattle (Reed et al., 2015; Bougouin et al., 2022), where models predicting N excretion from nutrient intake showed improved performance. The observation that DMI outperformed NI as a predictor variable in models predicting fecal N excretion in the current study could be attributed to several factors. Greater DMI can lead to greater digesta passage rate and secretion of digestive enzymes, ultimately leading to increased fecal DM output and fecal N excretion (Swanson, 1977; Reed et al., 2015). Alternatively, the use of DMI may lead to lower cumulative error compared to NI, which requires the measurement of N content in feed samples (Hristov et al., 2019). In contrast, urinary and manure N were best predicted from NI rather than DMI, which was expected as N intake is the major driver of urinary N excretion (Dijkstra et al., 2013) and urine is the major route of excretion of surplus N in beef cattle (Souza et al., 2021). It is well-documented that ruminal ammonia concentration increases in high CP diets (Souza et al., 2021). The ammonia not incorporated into microbial CP can be absorbed across the ruminal wall, entering the portal vein, and transported to the liver, where it is largely converted to urea (Batista et al., 2017). The synthesized urea can be eliminated by excretion in urine or recycled back to the gastrointestinal tract (Lapierre and Lobley, 2001). In fact, CP was not significant in the DC model predicting fecal N excretion, but it was in the DC model predicting urinary N excretion. In addition, when DC variables were combined with nutrient intakes with or without PF variables, CP had negative (models 5 and 8) and positive slopes (models 12 and 15) in more complex models predicting fecal and urinary N excretion, respectively. These findings corroborate previous studies showing that CP content is an important driver of urinary N excretion and determines the route of N excretion (Bougouin et al., 2022). For instance, Aarons et al. (2017) demonstrated that when dietary CP is high, there is a shift in the partition of N excretion with more N being excreted in urine than in feces. Reed et al. (2015) also reported a negative slope for CP content in a model predicting fecal N excretion in heifers and nonlactating cows. In this study, the NFC content was used as a proxy for energy content as it reflects the starch levels in the diet. The positive slope of NFC content in models predicting N excretion feces and manure could be explained by the positive correlation between energy levels (ME intake, KJ/d) and NI reported by Reed et al. (2015). However, the correlation between NFC and NI or CP content was weak and/or negative in this dataset (*r* = −0.01 and −0.31, respectively) and the fact that it was not significant in any of the models predicting urinary N excretion, which suggests that NFC may not be a good energy proxy, based on the current dataset. In contrast, NDF had a negative slope in models predicting manure N excretion and was not selected in any model predicting fecal or urinary N excretion. We hypothesized that diets with greater NDF concentration would have lower CP, which would decrease N excretion in urine (Dijkstra et al., 2013). The positive slopes observed for average BW and ADG can be explained by the linear and positive correlation between these variables with nutrient intakes and manure excretion (Weiss, 2004). In addition, it is expected that animals with accelerated growth rates receive nutrient-dense diets, which include greater levels of N compounds (Pinto and Millen, 2019). In agreement with previous studies (Yan et al., 2007; Waldrip et al., 2013; Bougouin et al., 2022), models derived based on PF (i.e., average BW and/or ADG) or DC variables alone had poor performance compared with univariate models using DMI or NI as predictors. However, in contrast with Bougouin et al. (2022), there was no improvement in model performance when PF variables were combined with nutrient intakes and DC variables.

## CONCLUSIONS

The models developed in this study could be used to predict N excretion in beef cattle over a diverse range of diets and animal genotypes in South America. The univariate model using DMI as a predictor is recommended for predicting fecal N excretion, whereas the univariate model using N intake is recommended for predicting urinary and manure N excretions. More complex models which include nutrient intakes and dietary composition might be more useful for decision-making when N excretion is a factor to be considered because they can allow simulations in N excretion with changes in diet composition with similar accuracy. Overall, manure N models resulted in lower error predictions than fecal and urinary N models and would be recommended when the route of N excretion is not of interest. Three extant equations evaluated in this study have the potential to be used in tropical conditions typical of South America to predict fecal N excretion with good precision and accuracy. However, none of the extant equations are recommended for predicting urine or manure N excretion because of their high RMSE, and low precision and accuracy. The models developed in this study could be used for national and international inventories when modeling manure ammonia and GHG emissions and in the evaluation of the effects of dietary interventions on N excretion from beef cattle and may be of interest for farmers, policymakers, and scientists.

## Supplementary Material

txae072_suppl_Supplementary_Materials

txae072_suppl_Supplementary_Figure_S1
