# Supplementary material for: Models to predict nitrogen excretion from beef cattle fed a wide range of diets compiled from South America
Source: Transl Anim Sci. 2024 May 9;8:txae072. doi: 10.1093/tas/txae072 (PMC11092400; doi:10.1093/tas/txae072)
Supplement: txae072_suppl_Supplementary_Materials [file txae072_suppl_supplementary_materials.docx]

**SUPPLEMENTAL MATERIALS**

**Models to predict nitrogen excretion from beef cattle fed a wide range of diets compiled from South America**

Vinícius C. de Souza†^,*^, Guilhermo F.S. Congio^‡^, João P.P. Rodrigues^#^, Sebastião C. Valadares Filho^††^, Flávia A.S. Silva^††^, Luciana N. Rennó^††^, Ricardo A. Reis^§^, Abmael S. Cardoso^‡‡^, Paulo H.M. Rodrigues^§§^, Telma T. Berchielli^§^, Juliana D. Messana^§^, Cecilia Cajarville^||^, Yury T. Granja-Salcedo^##^, Ana L.C.C. Borges^¶^, Gilberto V. Kozloski^†††^, Jaime R. Rosero-Noguera^‡‡‡^, Horacio Gonda^§§§^, Alexander N. Hristov^###^, Ermias Kebreab^†^

^†^Department of Animal Science, University of California, Davis, CA 95616, USA

^‡^Noble Research Institute LLC, Ardmore, OK 73401, USA

^#^Department of Animal Production, Animal Science Institute, Universidade Federal Rural do Rio de Janeiro, Seropédica, RJ 23897-000, Brazil

^††^Department of Animal Sciences, Universidade Federal de Viçosa, Viçosa, MG 36570-900, Brazil

^§^Department of Animal Science, Universidade Estadual Paulista, Jaboticabal, SP 14884-900, Brazil

^‡‡^Range Cattle Research and Education Center, University of Florida, Ona, FL 33865, USA

^§§^Department of Animal Nutrition and Production, Faculdade de Medicina Veterinária e Zootecnia, Universidade de São Paulo, Pirassununga, SP 13635-900, Brazil

^||^Department of Animal Production and Health of Production Systems, Animal Production Institute, Facultad de Veterinaria, Universidad de la República, San José 80100, Uruguay

^##^El Nus Research Center, Corporación Colombiana de Investigación Agropecuaria, San Roque, Antioquia 250047, Colombia

^¶^Department of Animal Science, Universidade Federal de Minas Gerais, Belo Horizonte, MG 31270-901, Brazil

^†††^Department of Animal Science, Universidade Federal de Santa Maria, Santa Maria, RS 97105-900, Brazil

^‡‡‡^Faculty of Agricultural Sciences, Universidad de Antioquia, Medellín, Antioquia 050034, Colombia

^§§§^Department of Animal Nutrition and Management, Faculty of Veterinary Medicine and Animal Science, Swedish University of Agricultural Sciences, Uppsala 75007, Sweden

^###^Department of Animal Science, The Pennsylvania State University, University Park, PA 16802, USA

^*^Corresponding author: [vinicius2042@hotmail.com](mailto:vinicius2042@hotmail.com)

**FIGURES**

**Figure S1.** Boxplot for fecal N excretion identifying outliers for the complete dataset.


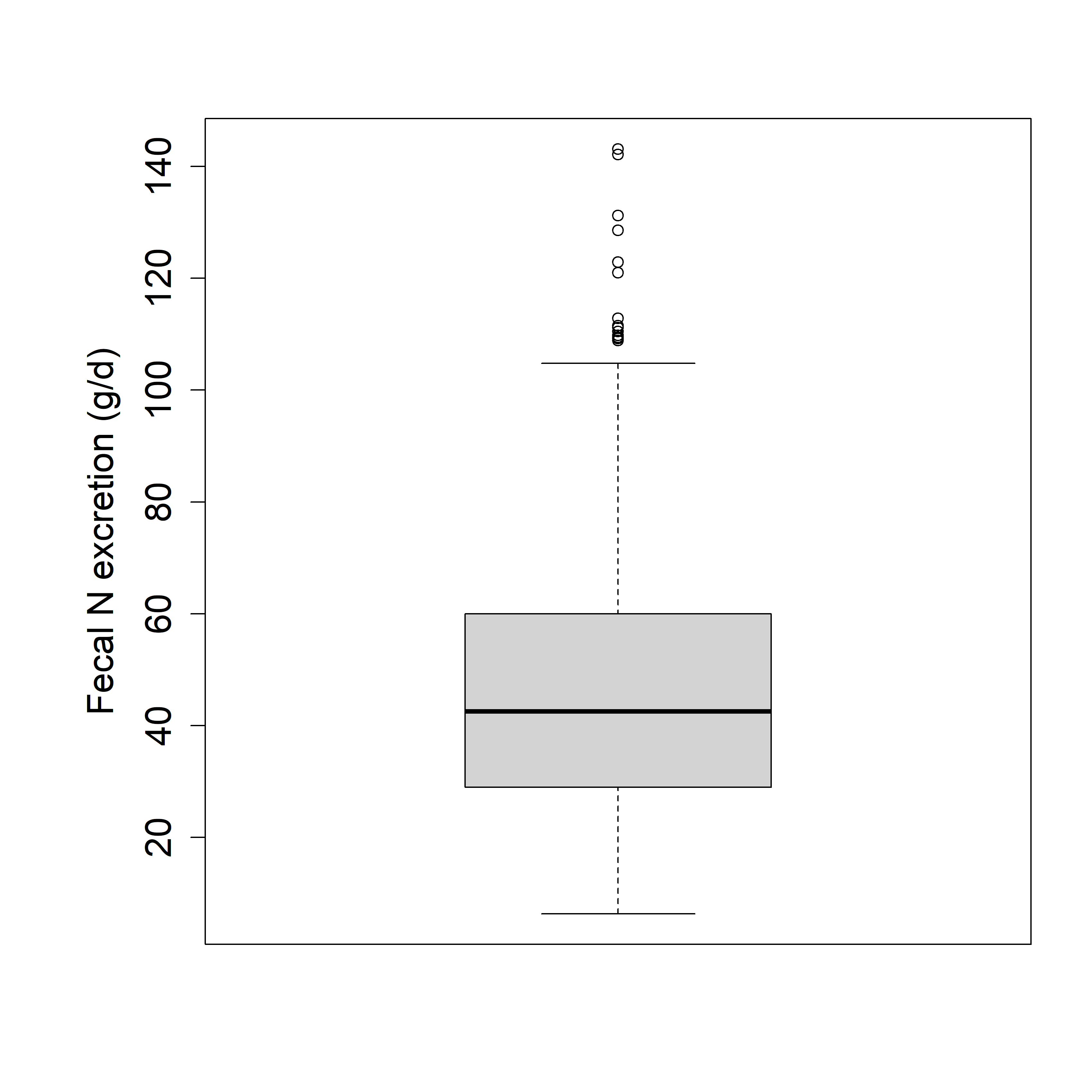


**TABLES**

**Table S1.** Studies used for model development and evaluation

| **Publication ID** | **Reference** | **Country** | ***n*^1^** |
| --- | --- | --- | --- |
| 1 | Escobar Restrepo, C.S. 2020. Balance energético y proteico, emisión de metano y requerimientos energéticos en ganado vacuno mestizo suplementado con recursos arbóreos y agroindustriales – Capítulo 3. PhD dissertation, Universidad de Antioquia, Medellín.  https://hdl.handle.net/10495/17495 | Colombia | 10 |
| 2 | Escobar Restrepo, C.S. 2020. Balance energético y proteico, emisión de metano y requerimientos energéticos en ganado vacuno mestizo suplementado con recursos arbóreos y agroindustriales – Capítulo 2. PhD dissertation, Universidad de Antioquia, Medellín.  https://hdl.handle.net/10495/17495 | Colombia | 14 |
| 3 | Chopa, F.S., L.B. Nadin, L. Agnelli, J. K. Trindade, H.L. Gonda. 2016. Nitrogen balance in Holstein steers grazing winter oats: effect of nitrogen fertilisation. Anim. Prod. Sci. 56:2039-2046.  doi: https://doi.org/10.1071/AN141007 | Argentina | 20 |
| 4 | Granja-Salcedo, Y.T., J. Duarte Messana, V. Carneiro de Souza, A.V. Lino Dias, L. Takeshi Kishi, L. Rocha Rebelo, T.T. Berchielli. 2017. Effects of partial replacement of maize in the diet with crude glycerin and/or soyabean oil on ruminal fermentation and microbial population in Nellore steers. Br. J. Nutr., 118:651-660.  doi:10.1017/S0007114517002689 | Brazil | 29 |
| 5 | Granja-Salcedo, Y.T., C. S. Ribeiro Júnior, R.B. de Jesus, A.S. Gomez-Insuasti, A.R. Rivera, J.D. Messana, R.C. Canesin, T.T. Berchielli. 2016. Effect of different levels of concentrate on ruminal microorganisms and rumen fermentation in Nellore steers. Arch. Anim. Nutr., 70:17-32.  doi: 10.1080/1745039X.2015.1117562 | Brazil | 32 |
| 6 | Rebelo, L.R., I.C. Luna, J.D. Messana, R.C. Araujo, T.A. Simioni, Y.T. Granja-Salcedo, E.S. Vito, C. Lee, I.A.M.A. Teixeira, J.A. Rooke, T.T. Berchielli. 2019. Effect of replacing soybean meal with urea or encapsulated nitrate with or without elemental sulfur on nitrogen digestion and methane emissions in feedlot cattle. Anim. Feed Sci. Technol., 257:114293.  doi: 10.1016/j.anifeedsci.2019.114293 | Brazil | 50 |
| 7 | Souza, V.C., J.D. Messana, E.D. Batista, K.L.G.C. Alves, E.C. Titgemeyer, A. Vaz Pires, M.V. Ferraz Junior, L.G. Silva, J.A. Negrão, V.E. Costa, M.J.G. Ganga, P.H.D. Colovate, T.T. Berchielli. 2021. Effects of protein sources and inclusion levels on nitrogen metabolism and urea kinetics of Nellore feedlot steers fed concentrate-based diets. J. Anim. Sci. 99:skab185. doi: 10.1093/jas/skab185 | Brazil | 33 |
| 8 | Camargo, K.D.V., J.D. Messana, L.G. Silva, Y.T. Granja-Salcedo, A.V.L. Dias, K.L.G.C. Alves, P.H. Gonçalves, W.A. Souza, R.A. Reis, T.T. Berchielli. 2022. Intake, metabolism parameters, and performance of growing beef cattle on pasture supplemented with different rumen undegradable protein with different amino acid profile. Anim. Feed Sci. Technol., 286:115258.  doi: 10.1016/j.anifeedsci.2022.115258 | Brazil | 27 |
| 9 | Orlandi, T., G.V. Kozloski, T.P. Alves, F.R. Mesquita, S.C. Ávila. 2015. Digestibility, ruminal fermentation and duodenal flux of amino acids in steers fed grass forage plus concentrate containing increasing levels of Acacia mearnsii tannin extract,  Anim. Feed Sci. Technol., 210:37-45.  doi: 10.1016/j.anifeedsci.2015.09.012 | Brazil | 16 |
| 10 | Ávila, S.C., G. Kozloski, T. Orlandi, M. Mezzomo, M., S. Stefanello. 2015. Impact of a tannin extract on digestibility, ruminal fermentation and duodenal flow of amino acids in steers fed maize silage and concentrate containing soybean meal or canola meal as protein source. J. Agric. Sci.153:943-953. doi:10.1017/S0021859615000064 | Brazil | 16 |
| 11 | Hoffmann, A., A.S. Cardoso, N.V.B. Fonseca, E.P. Romanzini, D. Siniscalchi, A. Berndt, A.C. Ruggieri, R.A. Reis. 2021. Effects of supplementation with corn distillers’ dried grains on animal performance, nitrogen balance, and enteric CH_4_ emissions of young Nellore bulls fed a high-tropical forage diet. Animal, 15:100155.  doi: 10.1016/j.animal.2020.100155 | Brazil | 24 |
| 12 | Berça, A.S., A.S. Cardoso, V.Z. Longhini, L.O. Tedeschi, R. M. Boddey, A. Berndt, R.A. Reis, and A.C. Ruggieri. 2019. Methane production and nitrogen balance of dairy heifers grazing palisade grass cv. Marandu alone or with forage peanut. J. Anim. Sci. 97:4625-4634.  doi: 10.1093/jas/skz310 | Brazil | 76 |
| 13 | Berça, A.S., A.S. Cardoso, N.V.B. Fonseca, D.P. Poppi, L.O. Tedeschi, I.R.C. Micheletti, W.R. Meireles, A.C. Ruggieri, R.A. Reis. 2023. Effect of diurnal feeding times and sources of energy supplementation to optimise rearing of F1 Angus × Nellore young bulls. Anim. Prod. Sci 63:579-595.  doi: 10.1071/AN22075 | Brazil | 32 |
| 14 | Leite, R.G. Recria de tourinhos nelore em pastos de capim marandu sob adubação nitrogenada – Capítulo 2. PhD dissertation. Universidade Estadual Paulista, Jaboticabal, 2021. 89p.  https://repositorio.unesp.br/server/api/core/bitstreams/454769b3-35e0-4bae-8802-f14f89d4d0ba/content | Brazil | 37 |
| 15 | Teobaldo, R.W., Y.T. Granja-Salcedo, A.S. Cardoso, M.T.L. Constancio, T.R. Brito, E.P. Romanzini, R.A. Reis. 2023. The impact of mineral and energy supplementation and phytogenic compounds on rumen microbial diversity and nitrogen utilization in grazing beef cattle. Microorganisms 11:810.  doi: 10.3390/microorganisms11030810 | Brazil | 25 |
| 16 | Silva, F.A.S., S.C. Valadares Filho, L.A. Godoi, B.C. Silva, M.V.C. Pacheco, D. Zanetti, P.D.B. Benedeti, F.F. Silva, T.L. Felix. 2020. Effect of duration of restricted-feeding on nutrient excretion, animal performance, and carcass characteristics of Holstein × Zebu finishing steers. Anim. Prod. Sci., 60:535–544. doi: 10.1071/AN18300 | Brazil | 24 |
| 17 | Silva, B.C., M.V.C. Pacheco, L.A. Godoi, G.A.P. Souza, N.V. Trópia, P. Pucetti, F.A.S. Silva, A.C.B. Menezes, L. N. Rennó, M.F. Paulino, J.P. Schoonmaker, S.C. Valadares Filho. 2021. Feed intake, nutrient digestibility, and selected rumen parameters in feedlot bulls fed diets with different feed additives. PLoS One, 16:e0259414.  doi: 10.1371/journal.pone.0259414 | Brazil | 34 |
| 18 | Menezes, A.C.B., S.C. Valadares Filho, M.V.C. Pacheco, P. Pucetti, B.C. Silva, D. Zanetti, M.F. Paulino, F.F. Silva, T.L. Neville, J.S. Caton. 2019. Oscillating and static dietary crude protein supply. I. Impacts on intake, digestibility, performance, and nitrogen balance in young Nellore bulls. Transl. Anim. Sci., 3:1205–1215.  doi: 10.1093/tas/txz138 | Brazil | 42 |
| 19 | Véras, R.M.L., S.C. Valadares Filho, J.A.G. Azevêdo, E. Detmann, M.F. Paulino, M.A. Fonseca, C.B. Sampaio. 2007. Níveis de proteína na dieta de bovinos Nelore de três condições sexuais: consumo, digestibilidades total e parcial, produção microbiana e parâmetros ruminais. R. Bras. Zootec., 36:1199-1211.  doi: 10.1590/S1516-35982007000500029 | Brazil | 42 |
| 20 | Silva, J.T., P. Pucetti, M.V.C. Pacheco, K.R. Oliveira, G.A.P. Souza , B.C. Silva, L.A. Godoi, H.M. Alhadas, A.C.B. Menezes, D.R. Andrade, G.S.F.M. Vasconcellos, T.S. Acedo, L.N. Rennó, F.F. Silva, S.C. Valadares Filho. 2022. Effect of vitamin blend supplementation on the ingestive, digestive, and ruminal parameters of Nellore cattle. Livest. Sci., 263:104986.  doi: 10.1016/j.livsci.2022.104986 | Brazil | 61 |
| 21 | Godoi, L.A. 2021. Corn processing in feedlot diets for Nellore cattle: intake, digestion sites and different techniques to estimate digestibility; and nutritional requirements and body composition of Nellore heifers by dual-energy x-ray absorptiometry or by carcass sections. PhD dissertation, Universidade Federal de Viçosa, Viçosa. | Brazil | 15 |
| 22 | Godoi, L.A., B.C. Silva, G.A.P. Souza, B.C. Lage, D. Zanetti, L.F. Costa e Silva, L.N. Rennó, M.F. Paulino, S.C. Valadares Filho. 2022. Influence of crude protein content and flint maize processing methods on the performance of early-weaning Nellore calves. J. Agric. Sci., 159:721-730.  doi: 10.1017/S0021859621001003 | Brazil | 15 |
| 23 | Perna Junior, F., R. Galbiatti Sandoval Nogueira, R. Ferreira Carvalho, E.C.O. Cassiano, P.H. Mazza Rodrigues. 2023. Use of tannin extract as a strategy to reduce methane in Nellore and Holstein cattle and its effect on intake, digestibility, microbial efficiency and ruminal fermentation. J. Anim. Physiol. Anim. Nutr. 107:89-102.  doi: 10.1111/jpn.13702 | Brazil | 14 |
| 24 | Borges, I.E. 2018. Combined use of monensin and nitrate as kinetic and ruminal fermentation manipulators to mitigate methane production in cattle. Master thesis, Universidade de São Paulo, Pirassununga.  https://www.teses.usp.br/teses/disponiveis/74/74131/tde-20062018-142234/en.php | Brazil | 32 |
| 25 | Solórzano Romero, L.A. 2019. Interaction between monensin and lipids addition in diets as manipulators of ruminal kinetics and fermentation for methane mitigation in ruminants. PhD dissertation, Universidade de São Paulo, Pirassununga.  https://www.teses.usp.br/teses/disponiveis/10/10135/tde-04122019-120802/en.php | Brazil | 32 |
| 26 | Tseu, R.J., F.P. Junior, R.F. Carvalho, G.A. Sene, C.B. Tropaldi, A.H. Peres, P.H.M. 2020. Rodrigues. Effect of tannins and monensin on feeding behaviour, feed intake, digestive parameters and microbial efficiency of nellore cows. Ital. J. Anim. Sci. 19:262-273.  doi: 10.1080/1828051X.2020.1729667 | Brazil | 31 |
| 27 | Carvalho, R.F. 2018. Essential oils as rumen fermentation modifier for enteric methane mitigation in ruminants. PhD dissertation, Universidade de São Paulo, Pirassununga.  https://www.teses.usp.br/teses/disponiveis/10/10135/tde-23112018-095349/en.php | Brazil | 16 |
| 28 | Assumpção, A.H.P.M. 2021. The individual and associated use of monensin, tannins and calcium nitrate in the feeding of cattle promote a reduction in the emission of methane gas. Master thesis, Universidade de São Paulo, Pirassununga.  https://www.teses.usp.br/teses/disponiveis/74/74131/tde-09082021-134509/en.php | Brazil | 24 |
| 29 | Sene, G.A. 2022. Strategic combination of feed additives aimed at manipulating ruminal kinetics and fermentation to increase energy efficiency and mitigate enteric methane emission and waste in ruminants. PhD dissertation, Universidade de São Paulo, Pirassununga.  https://www.teses.usp.br/teses/disponiveis/74/74131/tde-15082022-113656/en.php | Brazil | 28 |
| 30 | Britos, A., J.L. Repetto, C. Cajarville. 2018. Does it make a difference supplementing pasture silage with starchy concentrates or soyhulls on intake, digestion and rumen environment? Livest. Sci., 218:85-91.  doi: 10.1016/j.livsci.2018.09.021 | Uruguay | 24 |
| 31 | Santana, A., C. Cajarville, A. Mendoza, J.L. Repetto. 2016. Combination of legume-based herbage and total mixed ration (TMR) maintains intake and nutrient utilization of TMR and improves nitrogen utilization of herbage in heifers. Animal, 11:616-624.  doi: 10.1017/S1751731116001956 | Uruguay | 26 |
| 32 | Félix, A., J.L. Repetto, N. Hernández, A. Pérez-Ruchel, C. Cajarville. 2017. Restricting the time access to fresh forage reduces intake and energy balance but does not affect digestive utilization of nutrients in beef heifers. Anim. Feed Sci. Tech., 226:103-112.  doi: [10.1016/j.anifeedsci.2017.02.016](https://doi.org/10.1016/j.anifeedsci.2017.02.016) | Uruguay | 23 |
| 33 | Pancoti, C.G., 2015. Nutritional energy requirements from Gyr, Holstein and F1 Holstein × Gyr heifers. PhD dissertation, Universidade Federal de Minas Gerais, Belo Horizonte.  http://hdl.handle.net/1843/BUBD-9ZJGKD | Brazil | 18 |
| 34 | Ferreira, A.L., A.L.C.C. Borges, R.C. Mourão, R.R. Silva, A.C.A. Duque, J.S. Silva, A.S. Souza, L.C. Gonçalves, P.H.A. Carvalho. 2018. Energy partition, nutritional energy requirements and methane production in F1 Holstein × Gyr bulls, using the respirometric technique. Anim. Prod. Sci., 59:1253-1260.  doi: https://doi.org/10.1071/AN17432 | Brazil | 15 |
| 35 | Souza, A.S., 2016. Energy partition and methane production in Guzerá and Nelore heifers submitted to different nutritional plans. PhD dissertation, Universidade Federal de Minas Gerais, Belo Horizonte.  http://hdl.handle.net/1843/BUOS-ARCJKX | Brazil | 23 |
| 36 | Puseti, P. 2023. Concentrate inclusion levels in sorghum silage-based diets. PhD dissertation (In Preparation), Universidade Federal de Viçosa, Viçosa. | Brazil | 25 |
| 37 | Silva Júnior, J.M., L.N. Rennó, S.C. Valadares Filho, M.F. Paulino, E. Detmann, G.C.C. Menezes, T.S. Martins, R.M. Paula, J.P.P. Rodrigues, M.I. Marcondes. 2018. Evaluation of collection days and times to estimate urinary excretion of purine derivatives and nitrogen compounds in grazing Nellore cattle. Livest. Sci., 2017:85-91.  doi: 10.1016/j.livsci.2018.09.016 | Brazil | 25 |
| 38 | Silva Júnior, J.M., J.P.P. Rodrigues, S.C. Valadares Filho, E. Detmann, M.F. Paulino, L.N. Rennó. 2021. Estimating purine derivatives and nitrogen compound excretion using total urine collection or spot urine samples in grazing heifers. J. Anim. Physiol. Anim. Nutr. 105:861-873.  doi: 10.1111/jpn.13525 | Brazil | 25 |
| 39 | Silva, B.C., M.V.C. Pacheco, L.A. Godoi, H.M. Alhadas, J.M.V. Pereira, L.N. Rennó, E. Detmann, P.V.R. Paulino, J.P. Schoonmaker, S.C. Valadares Filho. 2020. Reconstituted and ensiled corn or sorghum grain: Impacts on dietary nitrogen fractions, intake, and digestion sites in young Nellore bulls. PLoS One, 15: e0237381.  doi: 10.1371/journal.pone.0237381 | Brazil | 25 |

^1^Number of observations.

**Table S2.** Studies where data of urine N excretion was excluded and reasons.

| **Publication ID** | **Reference** | **Reason for exclusion** |
| --- | --- | --- |
| 4 | Granja-Salcedo, Y.T., J. Duarte Messana, V. Carneiro de Souza, A.V. Lino Dias, L. Takeshi Kishi, L. Rocha Rebelo, T.T. Berchielli. 2017. Effects of partial replacement of maize in the diet with crude glycerin and/or soyabean oil on ruminal fermentation and microbial population in Nellore steers. Br. J. Nutr., 118:651-660.  doi:10.1017/S0007114517002689 | The authors excluded urine N excretion data from their publication due to problems in its determination. |
| 14 | Berça, A.S., A.S. Cardoso, N.V.B. Fonseca, D.P. Poppi, L.O. Tedeschi, I.R.C. Micheletti, W.R. Meireles, A.C. Ruggieri, R.A. Reis. 2023. Effect of diurnal feeding times and sources of energy supplementation to optimise rearing of F1 Angus × Nellore young bulls. Anim. Prod. Sci 63:579-595.  doi: 10.1071/AN22075 | Extremely low N excretion in urine, given N intake and BW (2.96 ± 2.81 % of N intake). |
| 15 | Leite, R.G. Recria de tourinhos nelore em pastos de capim marandu sob adubação nitrogenada – Capítulo 2. PhD dissertation. Universidade Estadual Paulista, Jaboticabal, 2021. 89p. | High variation in N excretion in urine within treatments. |
| 16 | Teobaldo, R.W., Y.T. Granja-Salcedo, A.S. Cardoso, M.T.L. Constancio, T.R. Brito, E.P. Romanzini, R.A. Reis. 2023. The impact of mineral and energy supplementation and phytogenic compounds on rumen microbial diversity and nitrogen utilization in grazing beef cattle. Microorganisms 11:810.  doi: 10.3390/microorganisms11030810 | This study tested the utilization of tannin sources, which can reduce N excretion. |
| 17 | Silva, F.A.S., S.C. Valadares Filho, L.A. Godoi, B.C. Silva, M.V.C. Pacheco, D. Zanetti, P.D.B. Benedeti, F.F. Silva, T.L. Felix. 2020. Effect of duration of restricted-feeding on nutrient excretion, animal performance, and carcass characteristics of Holstein × Zebu finishing steers. Anim. Prod. Sci., 60:535–544. doi: 10.1071/AN18300 | The authors excluded urine N excretion data from their publication due to problems in its determination. |
