# Supplementary figures and images for: Models to predict nitrogen excretion from beef cattle fed a wide range of diets compiled from South America
Source: Transl Anim Sci. 2024 May 9;8:txae072. doi: 10.1093/tas/txae072 (PMC11092400; doi:10.1093/tas/txae072)

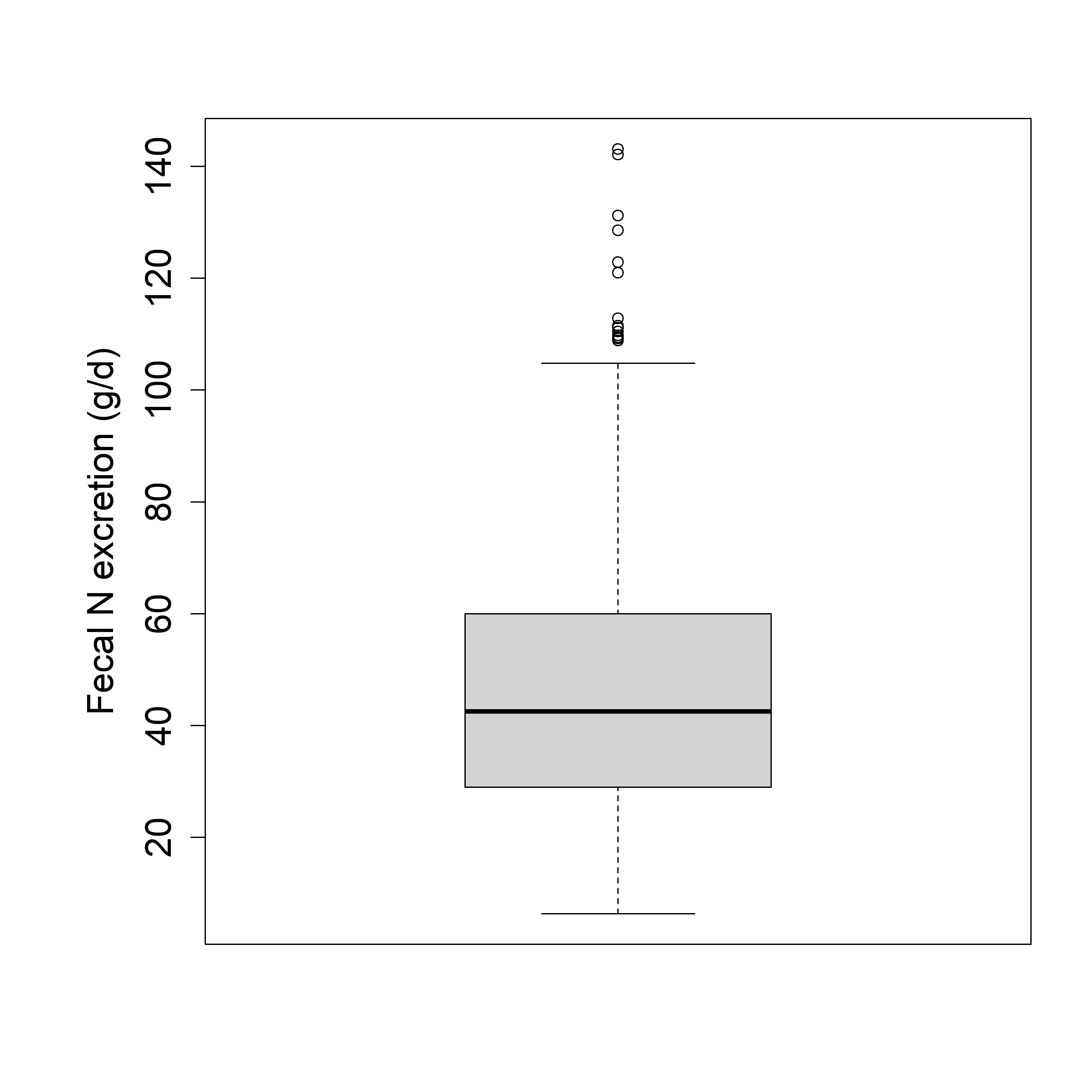

Supplement: txae072_suppl_Supplementary_Figure_S1 [file txae072_suppl_supplementary_figure_s1.jpeg]
